# Pityriasis Rubra

**Published:** 1881-02

**Authors:** James Nevins Hyde

**Affiliations:** Professor of Skin and Venereal Diseases, Rush Medical College


CHICAGO MEDICAL

JOURNAL & EXAMINER.

VOL. XLII.—FEBRUARY, 1881.—No. 2.

### Original Lectures.

ARTICLE I.

PITYRIASIS RUBRA.—Lecture delivered Nov. 22, 1880, at the Clinic, Rush Medical College. By James Nevins Hyde, M.D., Professor of Skin and Venereal Diseases, Rush Medical College. Reported by Dr. W. P. Verity.

*Gentlemen:*—Some of you were present in the neighboring necropsy theater of the Cook County Hospital, when I had the opportunity of examining the dead body of a man, with reference to whose case I desire to make some remarks this afternoon. Though requested by my colleague, in whose charge this patient had been, to examine the latter during life, I was unable to be present before the date of his death.

According to the statements of the hospital authorities, this patient had been a laboring man, was 32 years old, and a native of the State of New York, where he had lived up to within a very recent date. His father was killed during the late war, his mother died of some unknown disease at the age of 63, and he had three brothers and two sisters living, all in good health. An attack of scarlatina in childhood had resulted in excessive deafness which made it difficult to communicate with him ; and, later in life, he had suffered from typhoid fever without appreciable sequelæ. Two years ago, he considered himself in good health, but soon after this he noted the appearances, first upon his trunk and later upon his extremities, or from fifty to seventy-five, elevated cutaneous lesions which developed in succession. About eight months ago, there was extensive scaling of the surface of the body with an accompanying color change in the skin, which had been originally of a fair tint. When admitted to hospital, November 1, it resembled that of an octoroon, the nodules or "tubercles" referred to before, still appearing on its surface. So extensive was the exfoliation of the epidermis, that large handfuls of scales could be gathered from his bed each morning, the desquamation being most distinct on the dorsum, elbows and popliteal spaces. About the neck, hips, elbows and shoulders, fissures formed in the cuticle, which bled when irritated. A bed-sore formed during his illness, between the shoulder-blades, where several of these fissures had coalesced and where "tubercles" had existed. His emaciation was extreme and his weakness increased till locomotion became impossible. His tongue was thickly furred, his voice hoarse, appetite poor, and his sleep greatly disturbed by the distress occasioned by the itching of his skin. Evidence of disorder of the lungs, liver and spleen was wanting, but a systolic cardiac murmur was audible. The highest temperature attained during his stay in hospital was 100° F., and the most frequent pulse-beat 104 to the minute, though in both respects the normal standard was observed during the periods of comparative comfort. During the febrile exacerbations, there was considerable thirst.

He grew gradually weaker till an obstinate diarrhœa supervened, the fatal issue being announced by three separate convulsions in as many hours, on the afternoon of November 9. In the last of these he died, the relaxed sphincters permitting exit of the contents of the bladder and bowel, while the legs were flexed upon the thighs, and the thighs upon the abdomen.

A post-mortem section was not permitted, but the body was examined by me, almost as soon as cadaveric rigidity had occurred. The general color of the skin was very suggestive of the mulatto, and this was true of the entire surface. There was marked emaciation but no œdema of the lower extremities. The hair was thin, dry and short, and the expression of the face, still evident after death, pinched, anxious and worn. The entire surface of the body was, to a greater or less degree, covered with thin, dry, papery scales, grayish-white in color, which could be readily removed from the integument by passing the finger over them, and which, in places, were heaped up in lamellæ, especially over the scapulæ, hips and thighs. The skin did not seem to have been infiltrated to a marked extent, though the post-mortem evidences of such a condition would not have been perfectly appreciated. Here and there the sites of the fissures, described during life, were manifest, and the resulting bed-sore between the shoulders, was marked by a limited loss of tissue.

The so-called "tubercles," split-pea to marble sized elevations of the integument, were sparsely and unevenly distributed over the trunk and extremities, chiefly on the anterior and lateral aspect of the body, none whatever upon the face. When touched, these seemed to be, on pressure, moderately firm, but, detecting a softer point at the summit, I incised several, and thus gave exit to a quantity of fluid pus. The "tubercles" were really circumscribed subcutaneous abscesses, projecting upward through the skin proper. In some the subcutaneous cellular tissue had been involved to a greater extent than the size of the phlegmon would indicate when viewed superficially, for from several of smaller size, there flowed more than an ounce of matter.

The chief objective signs of disease, post-mortem, were, however, the singular pigmentation of the entire surface, and the universal exfoliation of the epidermis, in quantities sufficient to cover the hands passed over the surface.

An accurate post-mortem diagnosis of disease, is often as useful for the living as it is needless for the dead. In a case of disease of the skin, such a diagnosis is the more difficult to establish as the fluids which once circulated are at rest, and all the pathological processes ended. We are then not in the presence of an actual disease, but confronted with the imprints merely, of a disease which has vanished. In the case of several affections of the integument, as for example, the hyperæmiæ, even these imprints are removed with the life of the patient.

I think that the facts in the case described, justify us in believing that the patient, when living, suffered from pityriasis rubra, a disease whose extreme rarity, both in this country and abroad, renders its distinct recognition difficult and not unattended with a certain element of doubt. Since first described by Devergie, it has been the subject of observation and study by the late Prof. Hebra and Prof. Kaposi, of Vienna; by Professors Duhring, of Philadelphia; G. H. Fox, of New York; Drs. Guibout, of Paris; Jamieson, of Edinburgh, and others. If we accept the facts as reported by these authors, and by their aid interpret the utterances of the great master in dermatology, we must admit that the term pityriasis rubra should be extended to a wider range of phenomena, than the latter was at first disposed to allow. His three cases, all terminating fatally, were those of patients whose sole skin symptoms were, a crimson to pale-yellow tinting of the integument, this coloration being indistinguishable after death, and free exfoliation of large masses of dried epidermic plates, In all, cachexia was strongly marked. The itching was slight, and there was no infiltration in, nor secretion from, the skin. But in the case reported by Prof. Duhring, the skin *was* infiltrated. some fissures were noted, there was moderate itching, and decided involvement of the hair and nails. At the date of this report, a fatal result had not occurred. Under the title of "general exfoliative dermatitis,"* Jamieson describes a series of very interesting cases, which serve to extend still further our knowledge of the history of this disease and to aid in an appreciation of its variations from type. In the first of these, there was the same mulatto-like pigmentation, which was apparent, both before and after death, in the skin of the body examined by ourselves. There was also in Jamieson's patient, infiltration, extensive desquamation, and dusky redness of the surface, apparently complete recovery resulting. In his second case, fissures are described, and an accompanying diarrhœa. In a third, itching was a prominent symptom. In the last, there were successive

* *Ed. Med. Jour.*, April 1880, p. 879.

crops of imperfectly formed bullæ, which burst before becoming tense, the disease terminating fatally. Kaposi reports one case of recovery, and admits that the itching may be at times quite severe, and that fissures and infiltrations may be consequences of the process.

These cases, considered together, seem to me to justify the conclusion already stated, respecting the nature of the disease from which our patient suffered when alive. The question of diagnosis, however, involves the necessity of considering, in this connection, another disease, one also accompanied by epidermic exfoliation, and always, according to Hebra, of fatal issue, viz., pemphigus foliaceus. In the latter, crops of reddish or yellowish bullæ occur, usually not tense, their walls slightly corrugated, the injected vessels of the base showing through the thin layer of fluid contained in the bleb. The latter dries eventually into effete masses, said to resemble flaky pie-crust, and general exfoliation ensues. The health fails, as the disease progresses, and fresh crops of bullæ appear, diarrhœa supervening as a frequent epiphenomenon.

Had then this patient of ours, when living, the symptoms of this curious and equally rare disorder? I am inclined to think we should answer, both yes and no. Unquestionably, the appearance of the multiple subcutaneous abscesses suggests to the mind the ill-conditioned bullæ of pemphigus foliaceus, but then, in the form we have described, the roof-wall was constituted of the entire thickness of the epidermis. It was, thus, opaque, entirely obstructing the view of any vascular elements, were there such at the base of the lesion, and was superimposed upon an abundant thick pus, not the serum of the pemphigoid lesion. The circumscribed elevations of the epidermis, produced by the projection of these abscesses, were, moreover, of the color of the unaltered skin, and their creamy contents did not, by drying, furnish the material exfoliated from the surface, this last having evidently a much more superficial origin. The occurrence of multiple subcutaneous abscesses in any given form of disease, seems to be rather an accidental than an essential feature of any of them. Given a broken-down and depraved constitution, a profound change in the nutritive processes going on in the skin, severe or even moderate itching, associated with irritation of the surface by scratching with the finger-nails, and the complication named need not surely be regarded as at all extraordinary. We notice similar results in certain forms of eczema, in hereditary syphilis, severe acneiform lesions of the face, and in several of the benign affections of the scalp. In the present case, it is noteworthy that almost all of the abscesses occurred on those parts of the body most accessible to the hands.

At the same time, I am inclined to believe that the conclusions of Jamieson are entitled to consideration. Intermediate forms between typical pityriasis rubra and pemphigus foliaceus, should not surprise us. These words are, after all, but convenient titles under which we classify our facts as we gather them in the close observation of the several disorders of the integument. Jamieson regards the last mentioned disease as allied to, if not identical with the former, occurring in a more acute form than in that chronic superficial dermatitis, characterized by cuticular exfoliation, of long duration, rebellious to treatment, and frequently terminating fatally.

If there be such intermediate forms, the symptoms we have been considering should be assigned to such a category. The relatively brief time during which our patient suffered from his disease should not be forgotten, nor the febrile exacerbations revealed in his temperature record, nor yet the speedily manifested gravity of his symptoms, declared by the rapid failure of the powers of life. These all point to pemphigus foliaceus, while the symptoms of pityriasis rubra, as I have attempted to outline them, were no less prominent. The etiology and pathology of the two affections are equally obscure, and, considering the little that is known of the exact nature of each, we are justified by the facts in concluding that the two are expressions of a profound disturbance of the economy, possibly associated with lesions of other organs, of as great importance as the skin itself, the cutaneous manifestations approaching or departing from the type described by authors, as circumstances may determine. The nervous centers may be primarily at fault, but of this, in the absence of all observation and fact, we are not assured.

Few other diseases can be confounded with these, if we regard the feature of gravity imprinted on each, at some period in its course. I have heard that in this case a suggestion was made that the patient was leprous, and the marked pigmentation of the skin might have lent color to such a supposition. On hearing this, I remarked, at the time, that the so-called "tubercles" were not situated upon the face and forehead, where they are usually to be recognized in tubercular forms of leprosy; but the prompt discovery of the real nature of these abscesses dissipated at once the possibility of such an error. Leprosy in any form is, moreover, rarely fatal in such a relatively brief period of time, and when it furnishes a history of previously existing bullæ, we are tolerably sure to find distinct traces of these in variously shaped and sized atrophic patches, usually insensitive to the thrust of the lancet, and more pigmented in center or periphery than the surrounding integument. The hyper-pigmentation of leprosy is disposed with great irregularity, and rarely presents to the eye the uniformly-diffused, mulatto-like tint recognized in this case. The bullæ of anæsthetic leprosy are, in my observation, of deeper origin than those of pemphigus, and have a thicker roof-wall and less vascular floor. The contents, too, are usually limpid, and not decidedly purulent. The hoarseness of the voice also, in this case, lent some color to the leprous hypothesis; but you remember that we recognize this symptom in several other disorders accompanied by skin lesions, as, for example, syphilis, variola, scarlatina, lupus and carcinoma. Nothing but the discovery of leprous tubercles in the larynx would be sufficient, in the absence of other unmistakable symptoms, to establish the fact of leprosy. Hoarseness is sufficiently common in states of general adynamia, in emotional excitement, and even as a result of the internal use of an opiate. The debility of a patient affected with pityriasis rubra or pemphigus foliaceus, might be sufficient to produce this effect, but we can well understand how, in either case, a disease which is accompanied by an intestinal catarrh might display lesions of the mucous membrane of the respiratory tract distinguishable with the laryngoscope.

If this be indeed the grave picture of pityriasis rubra, what then is the nature of the cases, incorrectly designated by that name, of which we hear verbal accounts almost every year, the reports of which are not infrequently printed in the journals of medicine? Several such have been observed in this city, and of these a certain proportion have come under my own observation. I think that you will find the immense majority of them all to be either cases of psoriasis diffusa or of extensively-spread eczema squamosum. A few years ago I was called to see such a case in Mercy Hospital. The patient was a well-developed healthy male, who was shedding his epidermis freely from every portion of his integument. His bed-clothing was filled with scales, and the mere gentle passage of the hand over the surface of his skin was sufficient to induce a shower of pulverulent flakes. The integument beneath these was smooth, shining and crimson-hued, without a trace of fissure or sign of infiltration. The itching was by no means severe. It proved to be an aggravated form of psoriasis, the patient recovering rapidly under hospital treatment, showing characteristic palm-sized patches of the disease before he was dismissed.

Nearly of the same character was the case of a patient shown in this clinic last year, the slight difference between the two being due to the fact that, at the date of examination of the latter, the entire surface was not involved, though there was a history of such involvement. Recovery was prompt and without singularity. Another instructive case I observed in private practice, that of a young man, whose epidermis, when he came to me, was the source of an abundant dry, papery scaling, at every point of the surface of the body, the skin beneath being smooth, crimson-hued and very slightly infiltrated. The itching was of a mild character. He improved while wearing india-rubber under-garments, and taking arsenic, a remedy which, I may say in passing, has no beneficial effect upon pityriasis rubra, though administered in the largest doses and for the longest periods of time. This young man's disease relapsed, as is often the case in psoriasis, and the second efflorescence was in the form of perfectly typical lesions of psoriasis guttata. In neither attack was there the slightest interference with the general health.

It is proper to call your attention here to the fact that Dr. Tilbury Fox, though a careful observer and a dermatologist of experience, was unquestionably in error when he wrote that psoriasis never involved the entire surface of the body. My personal experience in these and a few other cases would have certainly convinced me of this, even if I had not seen a formal statement of the reverse of the proposition in the language of Prof. Hebra.

Personally, I cannot say that I have ever seen a case of squamous eczema which would suggest the disease we have under consideration, though such are described. A patient affected with universally diffused eczema was shown me last year by a student of this class, and the sick man was one of the most pitiable objects to be seen. His dull red skin was scaling extensively, but also weeping at innumerable points, the syrupy ooze drying here and there over the surface into characteristic eczematoid crusts. The infiltration was extensive, and the itching simply frightful. There is one tolerably pronounced feature of all cases of this class, which you will scarcely fail to notice. It is that the patient thus affected does not accommodate himself to his disease, as do the others. His skin is sore, tender, and exquisitely sensitive. He is apt to be almost as careful in the movements of his body and in the management of his eczematous limbs as if he were the subject of a fracture or a dislocation. While patients affected with this spurious "pityriasis rubra" (in other words, with exaggerated or diffuse psoriasis), will readily get out of the bed when requested to do so, will stand painlessly before their examiners, and assume such postures at the request of the latter, as best permit of a complete survey of the integumentary surface.

In pityriasis rubra, the prognosis is, you need not be told, unfavorable; and the treatment correspondingly unsatisfactory. It would be proper to apply bland oils and soothing unguents to the surface in order to protect the skin of the patient who is engaged in a labor aptly termed by Guibout "gigantesque." Such a course will merely assist in shielding from the inhospitable atmosphere the shivering victim who is involuntarily engaged in stripping himself of his epidermis. Internally the arsenical preparations should be avoided, and such tonic and roborant measures instituted, both as to diet and medicine, as are best calculated to sustain and invigorate the failing vital forces.

